# A content analysis of ‘junk food’ content in children’s TV programmes: a comparison of UK broadcast TV and video-on-demand services

**DOI:** 10.1093/pubmed/fdac067

**Published:** 2022-06-23

**Authors:** Alexander B Barker, Megan Parkin, Shreesh Sinha, Emma Wilson, Rachael L Murray

**Affiliations:** Department of Psychology, Nottingham Trent University, Nottingham, NG1 4FQ, UK; Academic Unit of Population and Lifespan Sciences, Faculty of Medicine and Health Sciences, University of Nottingham, Clinical Sciences Building, City Hospital, Nottingham, NG5 1PB, UK; Academic Unit of Population and Lifespan Sciences, Faculty of Medicine and Health Sciences, University of Nottingham, Clinical Sciences Building, City Hospital, Nottingham, NG5 1PB, UK; Academic Unit of Population and Lifespan Sciences, Faculty of Medicine and Health Sciences, University of Nottingham, Clinical Sciences Building, City Hospital, Nottingham, NG5 1PB, UK; Academic Unit of Population and Lifespan Sciences, Faculty of Medicine and Health Sciences, University of Nottingham, Clinical Sciences Building, City Hospital, Nottingham, NG5 1PB, UK; SPECTRUM Consortium, UK

**Keywords:** children, obesity, public health

## Abstract

**Objectives:**

Exposure to high in fat, sugar or salt (HFSS) food imagery is associated with unhealthy consumption, and subsequently obesity, among young people. We report and compare the results of two content analyses, one of popular children’s television channels in the UK and the other of a selection of children’s programmes available on video-on-demand (VOD) services.

**Methods:**

Content analysis of 3 days’ worth of programmes on two popular children’s television channels broadcast on UK television (CBeebies and Milkshake as well as a sample of children’s programmes available on the VOD platforms (Netflix and Amazon Prime) using 1-min interval coding.

**Results:**

In children’s television channels, HFSS content was seen in 181 episodes (36%) and in 417 intervals (13%) on terrestrial television, ‘Milkshake’ had a significantly higher proportion of broadcasts, which contained HFSS content than ‘CBeebies’. In VOD platforms, HFSS content was seen in 82 episodes (72% of the total number of episodes), across 459 intervals (19% of the total number of intervals), with no significant difference in the proportion of programmes containing HFSS content between Netflix and Amazon Prime.

**Conclusions:**

HFSS content is common in both popular UK children’s television channels and children programmes on VOD services and is likely having an effect on HFSS consumption in children. Legislative opportunities to prevent this exposure are being missed.

## Introduction

The United Kingdom (UK) is in the midst of an obesity epidemic.^1^ Across the UK, around one in four adults have obesity and a further one in three are overweight.^2–5^ Similar patterns are evident in children; over 20% of children in the final year of primary school in England have obesity and more than a third have obesity or overweight.^2^ These latter figures are of particular concern since children and adolescents with obesity typically become adults with obesity.^5–11^ Obesity is the third most important risk factor for chronic disease in the UK^12^ and in 2016 contributed to an estimated 70 000 premature deaths,^13^ costing the National Health Service $\sim$£6 billion annually.^14^ Preventing obesity is thus an important sociological and health priority.

Consumption of foods high in fat, sugar and/or salts (HFSS, or ‘junk food’) is a strong risk factor for obesity.^15^^,^^16^ Food choices in children are influenced by a range of factors,^17^ including exposure to depictions of HFSS foods in the media.^18–21^ Advertising, and particularly television advertising, is thus a major driver of HFSS consumption, particularly among children,^22–28^ and meets the criteria for a causal relationship when appraised against the Bradford Hill Causality Framework.^29^

The 2020 UK Government Obesity strategy^30^ recognizes the importance of advertising in driving HFSS consumption and proposes to reduce exposure to this by prohibiting HFSS (as identified by the Food Standards Agency nutrient profiling scheme^31^) advertising on television programmes broadcast before the 9 p.m. watershed, when children are likely to see them.^32^ Imposing a 9 p.m. watershed will likely reduce advertising exposure, but previous research into tobacco and alcohol content in UK terrestrial television programming^33^^,^^34^ shows that this content is often shown as a part of programme content, not just advertisements between programmes. A content analysis of the actual programme content of children’s television shows will allow us to determine whether current research is potentially missing content and whether current regulations may not be sufficient for protecting children from exposure to this content.

Furthermore, children’s viewing habits are changing. Video-on-demand (VOD) services, such as Netflix and Amazon Prime are becoming increasingly popular^35^ with children aged 5–16 now more likely to have watched a programme on VOD than on terrestrial TV channels such as BBC1 or ITV.^36^ Since 2015 VOD services based in the UK, including Amazon Prime Video, have been subject to Ofcom regulations similar to those covering terrestrial television channels. However, services based outside the UK are exempted from Ofcom regulations, instead subject to those of the country where they are registered.^37^^,^^38^ Netflix, which is based in Amsterdam,^39^ is subject to rules set out by the European Regulators Group for Audio-visual Media Regulators, including the European Audio-visual Media Services Directive,^40^ which applies controls on content deemed harmful to young people but does not comment on non-commercial portrayals of HFSS (or other harmful commodities) in programme content.^41^ Children’s programming on VOD services does not contain advertisements, but the HFSS imagery may therefore be more prevalent in content regulated outside the UK. Similar research has shown that tobacco and alcohol content is more common on VOD services than broadcast TV, regardless of content regulation on these services;^42^ however, little is known about HFSS content in programming from VOD sources.

HFSS advertising and content is a driver of HFSS consumption^22–28^ and is therefore a key public health issue; however, the research to date, and the current regulations aimed at restricting exposure to HFSS content, has focussed on advertisements^43^ and not the content of children’s programmes themselves. Furthermore, children’s programmes on VOD services may be covered by different regulations and may show more content than terrestrial television. We therefore report and compare the results of two content analyses, one of two popular children’s television channels in the UK and the other of a selection of children’s programmes available on VOD streaming sites.

## Methods

### Part one

We recorded 3 days’ worth of programmes (including advertisements) on the two most popular pre-school terrestrial (free-to-air) children’s television channels broadcast on UK television (^44^ CBeebies and Milkshake) from Wednesday 04 September 2019 to Friday 06 September 2019.

### Part two

We used the International Movie Database (IMDb) to determine the top 10 highest audience and reviewer rated original children’s programmes, and the top 10 highest rated other children’s shows (those not produced by the streaming service but simply available to view there) available on Netflix and Amazon Prime Video in April 2018. Children’s shows were determined as those that could be found in the children’s sections of each VOD platform. The selection process resulted in 40 programmes in total being chosen, 20 from each service. The series of each programme that aired in 2018 (or latest available if the series from 2018 was not available) were selected to be coded; the first, middle and last episode of each series was coded resulting in a sample of 120 episodes being included in the study.

To measure HFSS content in both parts of the current study, we adapted an interval coding method used extensively in previous studies^33^^,^^34^^,^^42^^,^^45–47^ adapted for coding HFSS content,^48^ coding the presence of audio-visual HFSS content in every 1-min interval of the programme, in the following categories.

#### Actual HFSS consumption

The actual consumption of an HFSS food on the screen coded according to Ofcom’s big 6^49^: confectionary, pre-sugared breakfast cereals, soft drinks, crisps and savoury snacks, fast food, pre-prepared convenience foods and other.

#### Implied HFSS consumption

HFSS content that was not consumed on-screen, but its consumption was implied (such as talking about eating cake without showing the cake). The category was split into the following groups: verbal (e.g. comment), non-verbal (e.g. behaviour—holding an HFSS item, chewing while holding item, etc.) and other.

#### Other HFSS

HFSS content being displayed but with no actual or implied consumption (such as cake being visible in the background). The category was split into the following groups: HFSS food packet, advertising, actual food shown, a brand associated with HFSS food but not shown in an advert, verbal reference but not implied, other.

#### HFSS Branding

Actual HFSS branding either shown on screen or mentioned. This was split into the following categories: HFSS packet/box, advertising/promotion (billboard/TV/Poster) and other.

Each occurrence of an HFSS food or content was measured once within each minute interval, but if it continued into the following minute, it was considered as a separate appearance. All branded or identifiable foods were cross checked against the Department of Health’s current Nutrient Profiling tool^50^ to ensure that they were classed as HFSS foods.

To ensure the accuracy and reliability in the coding method, 10% of data for each part of the study were coded separately by two authors. Data coding was completed in Microsoft Excel and, on completion, data were entered into IBM SPSS Statistics 24 for descriptive statistical analysis, as well as a χ^2^ analysis to compare the amount of content shown on broadcast TV and VOD services.

## Results

### Part 1—Broadcast children’s television channels

CBeebies ran from 06.00 to 19.00 every day on BBC, resulting in 13 h of content a day, 39 h in total. Milkshake ran from 06.00 to 9.15 on weekdays and 06.00 to 10.00 on the weekend on Channel 5, resulting in 11.15 h of Milkshake content. In total, 50.15 h of video was analysed in the content analysis.

In total, 505 separate broadcasts were coded (231 programmes, 53 adverts for other programmes, 158 scenes with channel presenters and 63 commercial adverts), totalling 3311 1-min intervals. 317 broadcasts were from CBeebies and 188 from Milkshake. HFSS content was seen in 181 broadcasts (36%) and in 417 intervals (13%) (Table 1).

**Table 1:** Number of programmes and intervals containing HFSS content on broadcast television

	Total (number containing/total)	CBeebies (number containing/total)	Milkshake (number containing/total)
Programmes *No of broadcasts* *No of Intervals*	72/231 (31%) 265/2826 (9%)	57/172 (33%) 198/2241 (9%)	15/59 (25%) 67/585 (11%)
Adverts for other programmes *No of broadcasts* *No of Intervals*	13/53 (25%) 16/59 (27%)	13/53 (25%) 16/59 (27%)	0/0 (0%) 0/0 (0%)
Channel presenter scenes *No of broadcasts* *No of intervals*	50/158 (32%) 63/275 (23%)	0/0 (0%) 0/0 (0%)	37/66 (56%) 43/85 (50%)
Commercial adverts *No of broadcasts* *No of Intervals*	46/63 (73%) 73/151 (48%)	No commercial adverts shown	46/63 (73%) 73/151 (48%)
Total *No of programmes* *No of Intervals*	181/505 (36%) 417/3311 (13%)	83/317 (26%) 234/2490 (9%)	98/188 (52%) 183/821 (22%)

Actual HFSS consumption was seen in 25 (5% of the total number of broadcasts) broadcasts and in 33 1-min intervals (1% of the total number of intervals). The most common form of HFSS consumption was confectionary (63% of intervals containing HFSS use) followed by pre-sugared breakfast cereals (17%), soft drinks (12%), crisps (4%) and fast food (4%). Implied consumption was seen in 27 programmes (5% of all broadcasts), across 33 intervals (2% of all intervals). Other HFSS content was seen in 164 broadcasts across both channels (33% of the total broadcasts) across 313 intervals (10% of intervals), with the most common content being food being shown on screen but not consumed (160 intervals, 51%). Seventy-seven intervals were due to the term ‘milkshake’, which is the name of the programming block seen on Channel 5.

A comparison between the amount of content on both channels showed that there was significantly more HFSS content shown in broadcasts on Milkshake than CBeebies (χ^2^ (1, N = 505) = 34.545, *P* = < 0.00); however, there was more actual (χ^2^ (1, N = 505) = 12.427, *P* = < 0.00) and implied consumption (χ^2^ (1, N = 505) = 13.718, *P* = < 0.00) shown on CBeebies. However, removing the channel name ‘Milkshake’ from the analysis shows that there was significantly more actual consumption (χ^2^ (1, N = 505) = 12.427, *P* = < 0.00) and implied use (χ^2^ (1, N = 505) = 13.718, *P* = < 0.00) shown on CBeebies (Table 2).

**Table 2:** Proportion (%) of programmes and intervals containing HFSS content on CBeebies and Milkshake

	CBeebies	Milkshake	Milkshake (excluding the term Milkshake)
**Any HFSS content**	26%	52%^a^	11%
**Actual HFSS consumption**	8%	1%^a^	1%^a^
**Implied HFSS consumption**	8%	1%^a^	1%^a^
**Other HFSS content**	21%	51%^a^	10%
**HFSS branding**	<1%	0%	0%

a Differences were statistically significant (*P* = < 0.05)

### Part 2—VOD services

In total, 114 episodes of children’s TV were coded (6 episodes were unavailable) from both streaming services; 54 episodes from Amazon Prime Video and 60 episodes from Netflix. The average run time of the episodes was 21 min. In total, 2448-min intervals were coded. HFSS content was seen in 82 episodes (72% of the total number of episodes), across 459 intervals (19% of the total number of intervals).

Actual HFSS consumption was seen in 40 episodes (35% of the total number of episodes), in 56 intervals (2% of the total number of intervals). The most common type of HFSS item seen being consumed in the programmes was confectionary, in 34 intervals (61% of all HFSS use references). Amazon Prime Video had 23 intervals containing HFSS consumption (11% of all HFSS references) over 17 episodes and Netflix 33 intervals (14% of all HFSS references) over 23 episodes. Implied HFSS consumption was seen in 83 intervals (18% of all HFSS references) over 37 episodes; 37 of these intervals were on Amazon Prime Video (45%) and 46 were on Netflix (55%). The majority of implied HFSS references were verbal (46 intervals, 55% of all implied HFSS references). Other HFSS references were seen in 406 intervals (88% of all intervals containing HFSS) over 81 episodes, with the majority of intervals containing HFSS products (288 intervals, 71% of the total other HFSS intervals). In total, there were four intervals containing HFSS branding (1% of all intervals containing HFSS references) over three episodes; three of these intervals were on Amazon Prime Video (*Nonni’s Biscotti, Marshmallow Fluff* and *Red’s True Barbecue*; 75%) over two episodes and the other interval was on Netflix (*Spam;* 25%). Three of the HFSS branding intervals contained a HFSS packet or box, two of these on Amazon and one on Netflix. The other HFSS branding reference fell into the ‘other’ category and was seen on Amazon Prime.

There was no significant difference in the proportion of programmes containing any HFSS content (χ^2^ (1, N = 114) = 3.013 *P* = 0.98), actual HFSS consumption (χ^2^ (1, N = 114) = .586 *P* = 0.56), implied HFSS consumption (χ^2^ (1, N = 114) = .374 *P* = 0.56), other HFSS consumption (χ^2^ (1, N = 114) = 2.256 *P* = 0.15) or branding (χ^2^ (1, N = 114) = .460 *P* = 0.60) between Netflix and Amazon prime Video (Table 3).

**Table 3:** Proportion (%) of programmes containing HFSS content on Amazon Prime Video and Netflix

	Amazon Prime Video	Netflix
**Any HFSS content**	80%	65%
**Actual HFSS consumption**	31%	38%
**Implied HFSS consumption**	30%	35%
**Other HFSS content**	78%	65%
**HFSS branding**	4%	2%

### Comparison between broadcast TV and VOD services

The proportion of programmes containing any HFSS content (χ^2^ (1, N = 619) = 49.569 *P* = < 0.00), actual (χ^2^ (1, N = 619) = 89.881 *P* = < 0.00) or implied HFSS consumption (χ^2^ (1, N = 619) = 73.733 *P* = < 0.00), any other HFSS content (χ^2^ (1, N = 619) = 57.878 *P* = < 0.00), and branding (χ^2^ (1, N = 619) = 8.579 *P* = 0.21) was significantly higher on VOD services than in broadcast television (Table 4).

**Table 4:** Proportion (%) of programmes containing HFSS content on Children’s UK broadcast television channels and Children’s VOD services

	Broadcast TV	VOD
**Any HFSS content**	36%	72%^a^
**Actual HFSS consumption**	5%	35%^a^
**Implied HFSS consumption**	5%	45%^a^
**Other HFSS content**	32%	71%^a^
**HFSS branding**	1%	3%^a^

a Differences were statistically significant (*P* = < 0.05)

## Discussion

### Main findings of the study

This study demonstrates that HFSS content is common in both popular pre-school children’s broadcast television in the UK and in children’s programmes on VOD services. The range of programmes on the TV and VOD services chosen for this study will appeal to a range of different ages in children (primarily pre-school on CBeebies and older children on VOD services) and although the mechanism of action on HFSS consumption via these programmes may differ according to the age of the child, exposure to HFSS content is known to be a major driver of consumption (43–49) affecting HFSS consumption in children and affecting eating habits throughout the lifespan.^5^^,^^51^ It is well recognized that children learn through observed behaviour, this has already been demonstrated in tobacco and alcohol use.^51–56^ It is likely that the same visual learning cues are encouraging children to develop food-choice behaviours aligned with screen-based observed behaviour through both advertising and editorial content.

### What is already known on this topic

Previous research has shown that HFSS content in the media has an effect on HFSS consumption, and children’s television programmes broadcast either on TV or VOD services are likely having an effect on HFSS consumption in children.

### What this study adds

To date, the research^43^ and subsequent regulation^30^ on HFSS content have focused on advertising, and this study shows that previous research has missed HFSS content in programmes and that programme content remains unregulated and may act as a way for brands to bypass the current regulation. Children are thus likely to continue to be exposed to HFSS content through programme content.

In regards to Cbeebies and Milkshake, which were shown on terrestrial television, the Ofcom regulations restrict advertisements for HFSS products being shown in programming made for children. This study suggests that HFSS content is being shown in programme content, potentially bypassing the current regulations which do not account for within-programming exposure. Despite being covered by the same Ofcom regulations, significant differences were found in the amount of content shown on two children’s television channels. Commercial channels featured higher levels of HFSS content overall, with a large proportion of this content being shown in commercial advertisements.

We found significantly more HFSS content on VOD programmes than terrestrial TV. As children are more likely to view VOD programmes than terrestrial TV (37), this represents a potential source of exposure to this content, which is currently unregulated. We found no difference between the proportion of HFSS content being shown on Amazon Prime Video or Netflix, despite differences in regulation. Amazon Prime Video is regulated by Ofcom and is subject to Ofcom’s regulations surrounding HFSS content being shown to children. Netflix is regulated by the European Audio-Visual Media Directive which does not specifically mention HFSS content, but has the overall goal of protecting young people from potentially harmful content. Although the Ofcom regulations only apply to HFSS advertising, which does not occur on VOD platforms, this current study highlights the fact that children are still being exposed to potentially harmful unregulated content through VOD services.

### Limitations of this study

This study is the first to examine the content of children’s terrestrial television programmes in the UK, previous studies have only focussed on advertisements,^22^^,^^24^^,^^25^^,^^43^^,^^57^ and the first to compare this to content with programmes on VOD services. This study used 1-min interval coding methods, established in previous studies on tobacco and alcohol content in the media.^33^^,^^34^^,^^42^^,^^45–47^^,^^58–62^ For logistic reasons we were able to code only a sample of days from broadcast TV and a sample of episodes from VOD services. We accept that our sample is small and therefore may not be representative of all children’s broadcast TV and VOD content, but this is an initial indication that children’s television programming represents a potentially harmful loophole in current advertising restrictions that warrants further research. We also recognize that in the absence of detailed viewing figures, we do not know the extent to which these channels and programmes were viewed by younger viewers. However, we know that these programmes are popular with younger viewers. In regards to terrestrial TV, Cbeebies shares 16.4% of 4–6 year old and 9.3% of children aged 0–3 years old viewing, and Milkshake was seen by 25% of 4–6 year old and 19% of children aged 0–3 in 2017.^44^ Children are also likely to have viewed children’s programmes on VOD services as children aged 5–16 are now more likely to have watched a programme on VOD than on terrestrial TV channels such as BBC1 or ITV.^36^ However, despite knowing that programmes on VOD are popular with young people, and in the absence of detailed viewing figures, we cannot know for certain whether these programmes are targeted at and / or viewed by young people. We recognize that the inclusion of the ‘Milkshake’ programming on Channel 5 could skew the results as this is the title of the schedule of programmes rather than a reference to HFSS being consumed, following the removal of this content from our analyses showed a significantly greater proportion of programmes on CBeebies showed actual and Implied HFSS consumption. The small sample of days chosen for this study was chosen due to limitations in coding resources, and we accept that this may have introduced bias to the study in the programmes showing different levels of content may be shown on other days of the week on terrestrial television and that the amount of content we saw may be due to chance.

This study highlights that research on children’s exposure to HFSS food content may have missed a large proportion of content by only considering advertisements and not the programmes themselves. Children’s programmes do contain HFSS content and previous research has shown that HFSS content, particularly advertising, in the media has a causal effect on children’s HFSS consumption through influencing children’s preference for these foods, and subsequently increasing the likelihood of children repeatedly asking their parents for these foods, so called ‘pester power’.^62^ Children’s television programmes broadcast either on TV or VOD services are likely having an effect on HFSS consumption in children. There is scope under the regulatory powers exercised by Ofcom to reduce the amount of HFSS content in programmes shown to children for UK broadcast television channels and VOD services based in the UK, such as Amazon Prime Video. We would urge Ofcom to consider the implications of allowing children to be exposed to HFSS content through programme content and to put regulations in place to prevent this from happening. We would also urge the Department for Health and Social Care to consider whether the current plan to ban ‘junk food’ adverts before the 9 p.m. watershed^30^ goes far enough to prevent exposure to HFSS content, and to consider HFSS content in programmes broadcast before this time.

## Declaration of Interests

None to declare.

## Funding

This work was supported by Cancer Research UK [C63710/A27908]. The funders had no role in the study design, data collection and analysis, decision to publish or preparation of the manuscript.

## Notes

**Alexander B. Barker,** Lecturer in Psychology

**Megan Parkin,** Medical Student

**Shreesh Sinha,** Medical Student

**Emma Wilson,** Associate Professor in Public Health

**Rachael L. Murray,** Professor in Population Health
